# Supplementary material for: The liver and muscle secreted HFE2-protein maintains central nervous system blood vessel integrity
Source: Nat Commun. 2024 Feb 3;15:1037. doi: 10.1038/s41467-024-45303-1 (PMC10838306; doi:10.1038/s41467-024-45303-1)

Supplementary Information:

Supplementary Methods:

DNA transfection & soluble ligand purification: Soluble HFE2, RGMa and 4IG was genetically tagged with six histidine residues at both N- and C-terminal and cloned into pSecTag2B expression vector (Invitrogen). DNA transfection and protein production protocol were inspired and modified from Aricescu et al [1]. Upon 90% confluency, human embryonic kidney (HEK293; ATCC Cat#CRL-1573)) cells were transfected with 60 µg of DNA / polyethylenimine (PEI) complexes (Sigma-Aldrich). Culturing media was changed from Gibco™ DMEM (Thermo Fisher) with 5% Gibco™ Fetal Bovine Serum (Thermo Fisher) and 1% Gibco™ Penicilin-Streptomycin (Thermo Fisher) to Gibco™ DMEM (Thermo Fisher) with 2% Gibco™ Fetal Bovine Serum after 6 hours following PBS wash. Transfected HEK cells were placed in the humidified incubator at 37ºC and 5% CO_2 environment for 3 days. On day 3, media was collected and centrifuged (1100 rpm for 5 min). To 50 mL of supernatant we added 1 mL of Ni-NTA Agarose Nickel-charged Resin (QIAGEN) for his-tag mediated protein binding. The mixture was incubated on a rocking bed with 50 rotations per minutes in 4ºC for 2 hours. After incubation, the solution mixture was poured into an empty Econo-Pac® Chromatography Column (Bio-Rad) to isolate the beads using gravity flow. The beads were washed three times with 50 mL of 5 mM imidazole (Sigma, I5513) + 500mM NaCl in 1xPBS. The proteins were eluted with 300 µL of 300 mM imidazole+500nM NaCl in 1xPBS. The elutes were placed in 12-14 kDa Fisherbrand™ Regenerated Cellulose Dialysis Tubing (Thermo Fisher) overnight in 4 ºC in 1xPBS (replaced with fresh for last 2-3 hours of incubation) to remove the imidazole in the solution.

Protein purity was assessed by running a 10% SDS gel followed by a 20-minute Coomassie Blue stain. This stain was then washed briefly in Coomassie de-staining solution. To confirm the presence of our protein of interest, a western blot was run using a 10% SDS gel. Prior to loading, 6X SDS loading dye was added to the protein sample and was heated at 95-100 ºC for 3-5 minutes in order to denature the protein. A Tris-glycine running buffer (25mM Tris, 192mM Glycine, 1% SDS) was used and the gel was run at 200V for 40 minutes. After completion, the gel was transferred to a nitrocellulose membrane (Bio-Rad) at 80 V for 1 hour at 4ºC. Following the transfer, the nitrocellulose membrane was blocked using a blocking solution consisting of 5% milk (powder) dissolved in PBS. The membranes were then incubated O/N at 4ºC in a PBST solution (5% blocking buffer with 0.1% tween in PBS) with the primary antibody (mouse monoclonal anti-His; abcam – G020). Membranes were then washed three times using PBST 0.1%, followed by a 1-2 hours incubation with the secondary antibody (list below) in PBST. Proteins were then visualized using the Odyssey infrared Imaging system.

Protein solution was concentrated using 10 kDa Amicon® Ultra-0.5 Centrifugal Filters (Sigma). The concentration of final protein product was measured following a 30-minute incubation at 37 ºC through optical absorption (spectrophotometer) at 560 nm using Pierce™ BCA Protein Assay Kit (Thermo Fisher) and aliquoted in the concentration of 1 µg/µL. Protein aliquots were stored in -20ºC.

Evans blue organ leakage assessment protocol: To assess vessel permeability in different organs, we conducted the Evans Blue Leakage Assessment protocol previously described by **Wick et al., 2018 [2].** Mice were anaesthetized with 2% Isofluorane and placed in a supine position. The ventral neck area of the mouse was shaved and a 1cm incision was made to the right of the ventral neck to expose the jugular vein. Mice were then injected with 200uL of 2% Evans Blue (ThermoFisher) via jugular vein and allowed to circulate for 30 minutes. Mice were then transcardially perfused with 20mL PBS and the following organs were surgically removed and weighed: heart, muscle, liver, kidney, lung, and brain. The organs

were then incubated in 400uL of formamide for 48 hours to extract the Evans blue. 50uL of the Evans blue-infused formamide from each sample was placed into a 96 well plate using pure formamide as the blank control. The optic density at 620nm of each well was measured and recorded. The OD_{620}/g wet weight of each organ sample was calculated.

Immunostaining of MS autopsy sections

Frozen brain tissue specimens acquired at autopsy from six patients with a definitive diagnosis of MS were obtained from the Human Brain and Spinal Fluid Resource Center, Veterans Affairs West Los Angeles Health Care Center., Los Angeles, CA, USA. Case 1- 53y old female, Case 2-53 years old female, Case 3 - 68 y old male, Case 4-62 y old female, Casse 5-47 y old female, Case 6 - 31 y old female. These brain tissue specimens were all autopsy samples from a tissue bank and were exempt from the IRB approval process according to the Department of Health and Human Services Office for Protection from Research Risk Code of Federal Regulations 45 CFR 46.101.6, Exemption No. HR-100201. Six control normal brains were obtained in form of normal brain tissue microarray from Biomax (Rockville, MD). Ages/sex of the patients (F, 55, F, 60, F 58, M, 42, F68, F, 62).

Immunostaining of brains of MS patients and control was performed as previously described [3]. In brief, 4-6 µm sections of human brain were fixed in acetone with 0.3% H_2O_2 for 10 mins at room temperature. The sections were blocked with 2.5% horse serum for 10 min and incubated with primary antibody anti-RGMa antibody (R&D Systems, Minneapolis, MN) overnight at 4 ºC. The following day, sections was incubated with secondary antibody followed by streptavidin and peroxidase complex (Vector labs). Sections were counterstained with hematoxylin (Sigma) and mounted with coverslip. All antibodies are listed in Table 1.

Western blot: Equal amount of proteins was loaded on 10% SDS-PAGE gel and separated by electrophoresis. The gel was transferred onto nitrocellulose membrane. Membrane was blocked with 5% BSA in PBS for one hour and incubated in primary antibodies diluted in 1% BSA overnight at 4 ºC. The following day, cells were washed with PBST (0.1% Tween) and incubated with species-appropriate secondary antibodies for one hour. Membrane was imaged using Odyssey® Classic Blot Imager (Li-COR). Relative abundance of the protein was normalized to the loading control using ImageJ. All antibodies are listed in Table1. Prior probing for Hfe2/RGMa in serum, albumin was removed using the Thermofisher albumin depletion kit following manufacturer instructions (Cat#85160).

Endothelial cell isolation & membrane extraction from mouse brains: To extract endothelial cells, ten mice brains were collected and homogenized together with Dounce tissue grinder in ice cold MCDB131 media (Dibco, 1935552). Homogenized tissue was resuspended in 15% 70 kDa dextran (Sigma, 44886) and centrifuge at 10,000 g for 30 mins. The pellet was digested in 1 mg/mL collagenase & dispase (Roche, Colldisp-ro) in 37 ºC for 2 hours. The digested product was resuspended in 45% Percoll and centrifuged at 20,000 g for 10 mins. Isolated endothelial cells at the top layer were collected and washed with ice cold PBS. Isolated cells were then place in membrane HB buffer (10 mM HEPES, 25 mM KCl, 5 mM $MgCl_2$) and resuspended thoroughly. Sucrose gradient was created using 50% sucrose overly with 5% sucrose. Resuspended cells in HB buffer was added on top of 5% sucrose and centrifuged at 28,000 rpm for 10 mins. Membrane in the middle phase was removed and concentration was measured using a Pierce™ BCA Protein Assay Kit. Equal amount of membrane was used for Western blot.

Endothelial cell extraction and FACS isolation: Endothelial cell extraction protocol was adopted and modified from Chen et al. 2004 [4]. Mice were euthanized by cervical dislocation and brains were collected into the DMEM supplemented with 5% FBS. Brains were then minced into small pieces of ~1x1mm in

size, homogenized by 10mL syringe and transferred to 15-mL falcon tube. Homogenized tissues were centrifuged at 300g at 4 °C for 5 minutes and pellets were resuspended in 15mL of collagenase/dispase solution (split into 3 tubes). Solution containing cell pellets were rotated for 30 minutes at 37 °C to be digested and pelleted again in another 300g at 4 °C centrifugation. Pellets were then triturated via pipetting in 1ml of 2% FBS in PBS containing 50ul of DNase per tube. To remove myelin and debris from the solution, 4ml of 22% Percoll per tube was added to tubes, and solution containing digested tissues were added on top of Percoll layer. After 10 minutes at 560g at 4 °C, pellets were collected and resuspended in HBSS/BSA/glucose buffer to be ready for immunostaining and flow cytometry fluorescence activated cell sorting (FACS).

Antibodies (see Table1) were added to resuspended cells (FACS sample – 400ul, each control – 100ul each) at the following concentrations: CD31 (PE, 1:50, 4μg /ml), CD41 (APC, 1:200, 1μg/ml), SYTOX blue dead cell stain (ThermoFisher S34857, 1:1000). Treated cells were incubated for 15 minutes on ice covered from light and washed with HBSS/BSA/glucose buffer, then sent to flow cytometry facility.

Flow Cytometry and Intracellular Cytokine Staining

Mice were euthanized at day 10 post-induction of EAE. Freshly isolated spleens and draining caudal lymph nodes (LN) were pressed using a syringe plunger through a 70μm cell strainers and washed twice using 2mL of 2% FBS in complete RPMI-1640 medium (Invitrogen) supplemented with 2-β Mercaptoethanol, L-glutamine, p/s, and non-essential amino acids. Single-cell suspensions were lysed with 7mL of red blood cell (RBC) lysing buffer (Life Technologies, Cat# AM8728) and centrifuged at 700 rcf for 5 mins. After washing three times with 2% RPMI-1640 medium, cells were counted and cultured as described below or proceeding to cell surface staining.

The expression of cell-surface markers and cytokine expression on splenocytes and LN cells were characterized by flow cytometry. For analysis of naïve cells, 5x105 RBC depleted splenocytes and LN cells were plated in 96-well V-bottom plates (Starstedt) and blocked with 50μl of 1% mouse IgG in 2%FBS PBS supplemented with azide (PFN3) for 20 mins at 4°C. Cells were then stained with the following primary conjugated antibodies for 30 mins on ice.

All antibodies were re-suspended in PFN3. The list of all antibodies can be found in Supplementary Table 2.When required, cells were washed twice with 100μl of PFN3 and stained with secondary SA-APC, SA-APC-Cy7, SA-PeCy7, or SAPercp-Cy5.5 for 30 mins on ice. Cells were then washed twice with PFN3 and resuspended in a PFN3 solution containing propidium iodide (PI) before proceeding to FACS acquisition.

For examination of intracellular cytokine production, 0.5x106 RBC depleted splenocytes and LN cells were plated in 96-well flat bottom plates (BD Biosciences) in 10% FBS in complete RPMI-1640 in the presence of 0.5μg/mL phorbol 12-myrisate 13-acetate (PMA), 0.5μg/mL Ionomycin, and Golgistop (BD Biosciences) for 4 hrs. Cells were subsequently washed in PBS and stained with Near-Infrared Live Dead (Life Technologies) as described in the manufacturer's protocol. Cells were then washed twice in PFN3 and stained for cell surface markers as described previously. Cells were then 43 fixed with 100μl of Cytofix/Cytoperm solution as described in the manufacturer's protocol. For intracellular staining, cells were then stained with anti-IL-17A, and antiIFN-γ for 30 mins on ice, washed twice, and resuspended in PFN3 before proceeding to FACS acquisition.

Nonspecific background staining was determined by using fluorochrome-matched isotype antibodies and fluorescence minus one control. Samples were acquired in a BD LSRII or FACS CantoII cytometer (BD

Biosciences). For each experiment, at least 100,000 live events were acquired and analyzed using Flowjo software (Tree Star Inc.).

Statistical analysis: Statistical analysis was done using GraphPad Prism software (https://www.graphpad.com/scientific-software/prism/). Unless otherwise stated, unpaired Student t-test was used to compare significances between two groups. Multifactorial analysis of variance (ANOVA) with post-hoc Bonferroni test was used to compare significances between two or more groups in cases where a single complementary p-value could not be computed. $P<0.05$ was considered statistically significant.

Retinal explants outgrowth assay and axonal staining. Poly-L-Lysine (SIGMA; 10µg/ml) coated glass coverslips were treated with Laminin (Invitrogen; 10µg/ml) and RGMa (5µg/ml) and incubated for 3hrs at room temperature. Temporal retinal explants were cultured on protein-coated surfaces for 18h. Treatment was done adding purified HFE2/RGMc protein at the indicated concentration right after seeding the explants on the RGMa/Laminin substrate. Explants were then fixed with 4% PFA and stained with Alexa488-phalloidin. Fiber length was quantified using Image Pro 5.0.

YAP Activity Assessment via Luciferase. In this experiment, we harnessed the dual-luciferase assay to gauge YAP activity within bEnd3 cells following transfection with Lipofectamine 3000 (Invitrogen). To specifically assess YAP activity, we introduced a plasmid containing the YAP gene and firefly luciferase reporter to the cells and treated with PBS of 10ug/mL HFE2 protein for 18 hours, 2 days post-transfection. After treatment, the bEnd3 cells were lysed, allowing us to sequentially measure the activity of the firefly luciferase using the Dual-Luciferase® Reporter Assay System (Promega) following the manufacturer's protocol. The luminescent signal served as a direct indicator of YAP expression and activity within the bEnd3 cells. To ensure the accuracy of our results, we employed *Renilla*-luciferase as an internal control to normalize for potential variations in transfection efficiency and cell viability.

Hanging wire (strength) test: Male and female adult mice (18-28 g) were freely suspended for 3 minutes from a steel wire (2 mm diameter, 55 cm long, and elevated 35 cm above soft bedding) with replacement after falling or reaching the end of the wire. Occurrences of falls and reaching events were recorded. Mice were handled by the tail and allowed to grasp wire with their forelimbs during placement.

Primer specificity	Forward (5' to 3')	Reverse (5' to 3')
Hfe2 (LoxP)	GAATGGCTTCCTTCCATCAA	ATCTTCAAAGGCTGCAGGAA
Albumin-Cre	TGCAAACATCACATGCACAC	TTGGCCCCTTACCATAACTG
ACTA1-Cre	GCGGTCTGGCAGTAAAAACTATC	GTGAAACAGCATTGCTGTCACTT
Neogenin	TGGGTAGCTCGATCCTCAGT	TATGTGCTCGCCAAAAACAA
Tie2-Cre (LoxP)	CGCATAACCAGTGAAACAGCATTGC	CCCTGTGCTCAGACAGAAATGAGA

Supplementary Table 1: Primers used for genotyping.

Antibody	Species	Clone	Company	Dilution
CD5	Rat	53-7.3	BioLegend	1:200
CD3	Rat	145-2C11	BioLegend	1:200
CD4	Rat	GK1.5	BioLegend	1:200
CD8	Rat	53-6.7	BioLegend	1:200
CD19	Rat	6D5	BioLegend	1:200
CD11b	Rat	M1/70	BioLegend	1:200
CD11c	Hamster	N418	BioLegend	1:200
CD80	Hamster	16-10A1	BioLegend	1:200
CD86	Rat	GL-1	BioLegend	1:200
CD44	Rat	IM7	BioLegend	1:200
MHC II	Rat	M5/114.15.12	BioLegend	1:200
CD62L	Rat	MEL-14	BioLegend	1:200
ICAM-1 (CD54)	Rat	3E2	BioLegend	1:200
IL-17A	Rat	TC11-18H10.41	BioLegend	1:200
IFN-γ	Rat	XMG1.2	BioLegend	1:200

Supplementary Table 2: Flow cytometry antibodies

References:

1 Aricescu, A. R., Lu, W. & Jones, E. Y. A time- and cost-efficient system for high-level protein production in mammalian cells. *Acta Crystallogr D Biol Crystallogr* **62**, 1243-1250, doi:10.1107/S0907444906029799 (2006).

2 Wick, M. J., Harral, J. W., Loomis, Z. L. & Dempsey, E. C. An Optimized Evans Blue Protocol to Assess Vascular Leak in the Mouse. *J Vis Exp*, doi:10.3791/57037 (2018).

3 Rus, H. *et al.* The voltage-gated potassium channel Kv1.3 is highly expressed on inflammatory infiltrates in multiple sclerosis brain. *Proc Natl Acad Sci U S A* **102**, 11094-11099, doi:10.1073/pnas.0501770102 (2005).

4 Chen, D., Zhao, M. & Mundy, G. R. Bone morphogenetic proteins. *Growth Factors* **22**, 233-241, doi:10.1080/08977190412331279890 (2004).

Supplementary Figures:

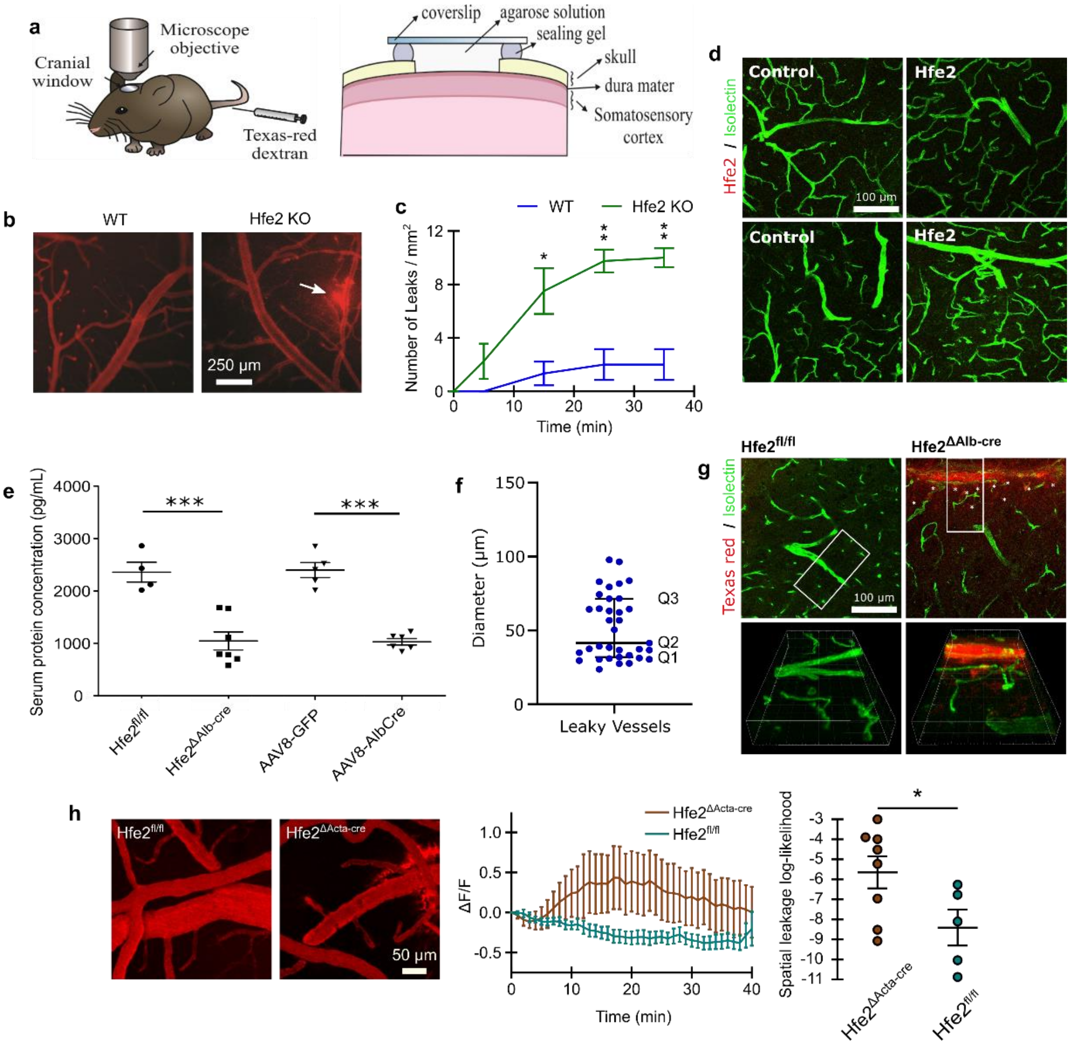

Supplementary Fig. 1 | Fluorescent imaging of BBB disruption in *Hfe2* KO mice. a, A schematic of widefield imaging procedure and cranial window. **b,** Representative widefield BBB leakage patterns of TR-dextran in wild type and *Hfe2* KO mice. **c,** Quantification of the number of BBB leaks observed in wild type and *Hfe2* KO mice over time (mean ± s.e.m., unpaired two-tail t-test, replicates WT n=3 and *Hfe2* KO n=4). **d,** Absence of Hfe2 within brain tissue of WT mice; confirmed with confocal imaging. Control images correspond to secondary anti-body incubation without HFE2 primary antibody. **e,** Serum concentration of HFE2 quantified with ELISA in (mean ± s.e.m.; unpaired two-tail t-test; $Hfe2^{fl/fl}$ n=4, $Hfe2^{\Delta Alb\text{-}cre}$ n=7, AAV8-GFP n=5, and AAV8-AlbCre n=6). **f,** Diameter of vessels where TR-dextran leakage was observed. **g,** Confocal image of TR-dextran accumulation with endothelium counterstain. **h,** Representative *in-vivo* multiphoton images of TR-dextran at 40mins time-point (scale bar, 50 μm). The normalized extravascular fluorescence (ΔF/F) intensity was plotted over time (mean ± s.e.m.; unpaired two-tail t-tests) and the groups differences were assessed with spatial leakage log-likelihood (repeated measures factorial ANOVA rendered as approximate unpaired two-tail t-test; $Hfe2^{fl/fl}$ n=5, $Hfe2^{\Delta Acta\text{-}cre}$ n=8). *P<0.05, **P<0.01, ***P<0.001. Source data are provided as a Source Data file (includes exact p-values).

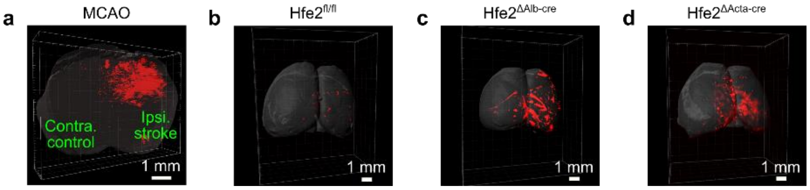

Supplementary Fig. 2 | Light sheet imaging of BCB breakdown associated with MCAO and Hfe2 ablation. a, 3D light sheet image of *Hfe2*$^{fl/fl}$ mouse leakage profile after injection and circulation of 70kDa dextran conjugated dye for 1 hour followed by iDISCO clearing. **b,** 3D light sheet image of *Hfe2*$^{fl/fl}$ mouse leakage profile after injection and circulation of 70kDa dextran conjugated dye for 1 hour followed by iDISCO clearing. **c,** 3D light sheet image of *Hfe2*$^{\Delta Alb\text{-}Cre}$ mouse leakage profile after injection and circulation of 70kDa dextran conjugated dye for 1 hour followed by iDISCO clearing. **d,** 3D light sheet image of *Hfe2*$^{\Delta Acta\text{-}Cre}$ mouse leakage profile after injection and circulation of 70kDa dextran conjugated dye for 1 hour followed by iDISCO clearing.

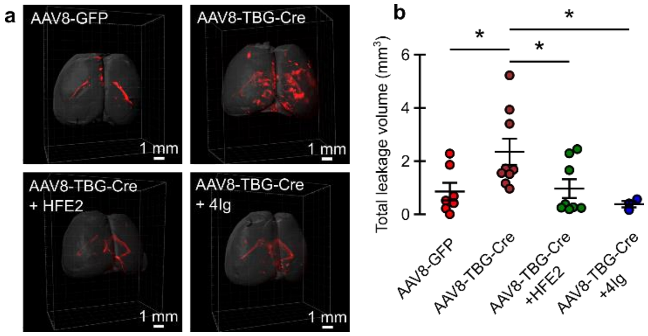

Supplementary Fig. 3: Treatment with HFE2 and 4Ig rescue blood vessel integrity in *Hfe2* liver KO mice. a, 3D full brain rendered representative light-sheet images of TR-dextran deposition in iDISCO cleared brains of *Hfe2*$^{fl/fl}$ mice injected with AAV8-GFP and AAV8-TBG-Cre viral injected mice treated with or without HFE2 or 4Ig protein treatment (scale bars, 1mm). **b,** Quantification of total leakage volume (mean ± s.e.m.; unpaired two-tail t-test; AAV8-GFP n=7, AAV8-TBG-Cre n=9, AAV8-TBG-Cre + HFE2 protein treatment n=8, AAV8-TBG-Cre + 4Ig protein treatment n=3). Source data are provided as a Source Data file (includes exact p-values).

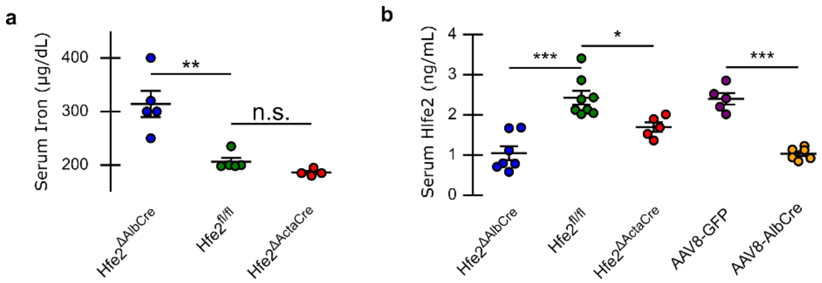

Supplementary Fig. 4 | *Hfe2*-muscle specific KO mice have normal serum iron and reduced HFE2 levels. a, Serum iron levels in liver- and muscle-specific knock-outs quantified by optical density using iron detection kit (VWR, 75878-098) (mean ± s.e.m., unpaired two-tail t-test, replicates *Hfe2*$^{\Delta Alb\text{-}cre}$ n=5, *Hfe2*$^{fl/fl}$ n=5, and *Hfe2*$^{\Delta Acta\text{-}cre}$ n=4). **b,** Reduced serum HFE2 in liver- and muscle-specific knock-out quantified with ELISA kit (R&D, MRGMCO) (mean ± s.e.m.; unpaired two-tail t-test, replicates *Hfe2*$^{\Delta Alb\text{-}cre}$ n=7, *Hfe2*$^{fl/fl}$ n=8, *Hfe2*$^{\Delta Acta\text{-}cre}$ n=5, AAV8-GFP n=5, and AAV8-AlbCre n=6). *P<0.05, **P<0.01, ***P<0.001. Source data are provided as a Source Data file (includes exact p-values).

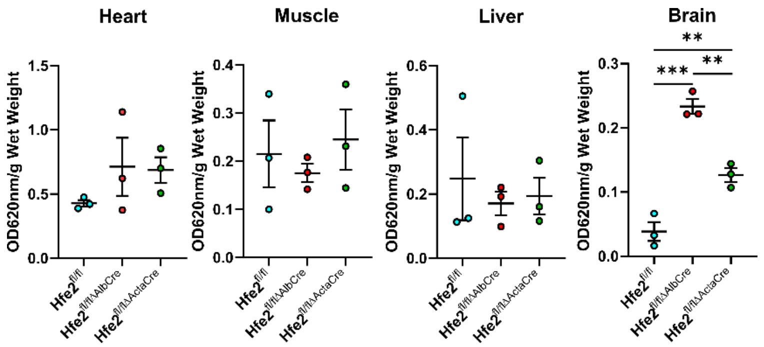

Supplementary Figure 5 | Evans Blue extravasation in organs: Evaluation of vascular leakage in organ blood vessels using Evans Blue. Vessel permeability assessment in *Hfe2*$^{fl/fl}$, *Hfe2*$^{\Delta Alb\text{-}Cre}$, and *Hfe2*$^{\Delta Acta\text{-}Cre}$ mice following a 30-minute injection of Evans Blue via the jugular vein. Heart, lung, muscle, and brain tissues were perfused, extracted, and placed in formamide for 48 hours to detect variations in vessel permeability between the groups. The quantification of leakage (OD620/g wet weight) in each organ is depicted (mean ± s.e.m.; unpaired two-tail t-test; *Hfe2*$^{fl/fl}$ n=3, *Hfe2*$^{\Delta Alb\text{-}Cre}$ n=3, and *Hfe2*$^{\Delta Acta\text{-}Cre}$ n=3). *P<0.05, **P<0.01, ***P<0.001. Source data are provided as a Source Data file (includes exact p-values).

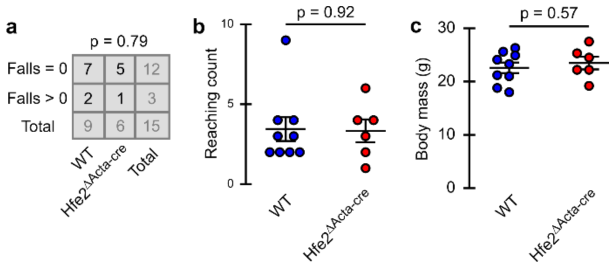

Supplementary Fig. 6| Hanging wire strength test for muscle knock-out. a, Contingency table analysis of falling events from hanging wire test for *Hfe2*$^{\Delta\text{Acta-cre}}$ mice and their litter mate (Acta-cre negative) controls. **b,** Reaching count (mean ± s.e.m.; unpaired two-tail t-test) for same mice during the hanging wire test. No statistical differences were found between groups with respect to the number of falling events and the reaching count. **c,** The body mass of mice (mean ± s.e.m.; unpaired two-tail t-test) were similar between the two groups indicating strength differences were fairly assessed by this test. Source data are provided as a Source Data file (includes exact p-values).

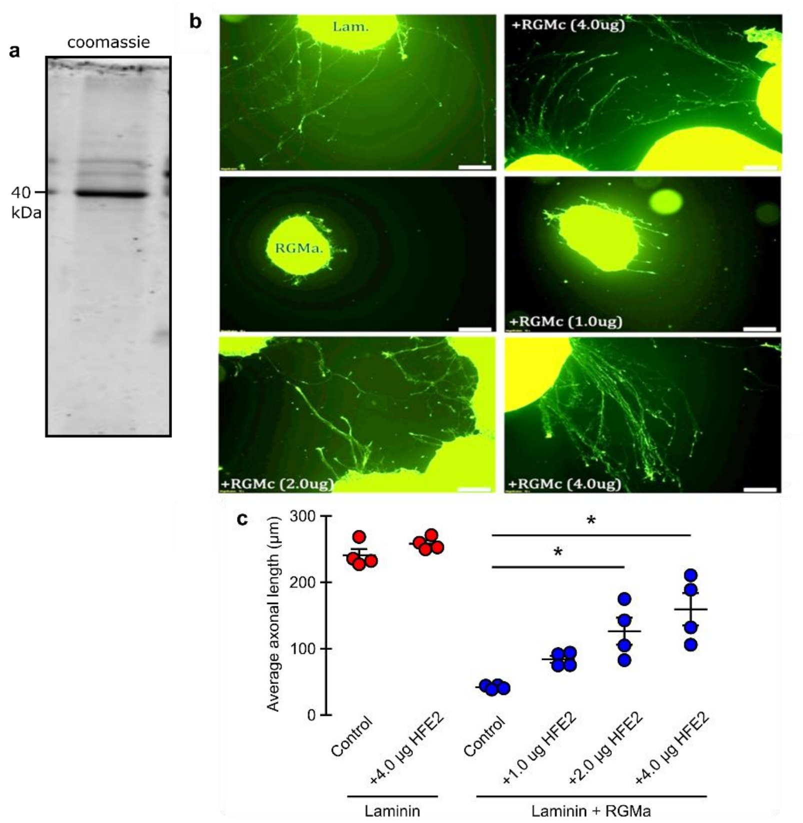

Supplementary Fig. 7 | Effect of HFE2 (RGMc) on axonal grown in retinal explants. a, Coomassie stain of HFE2/RGMc purity. **b,** Laminin fluorescent labeling of neurons. Scale Bar, 200µm. **c,** Quantification of axonal length from laminin labelling. Retinal explants were grown on laminin with and without RGMa. RGMc/HFE2 was added to the medium at various concentrations (PBS was control). Axons are shorter on laminin + RGMa when compared to Laminin on its own. When RGMc is added to the medium axons appear longer and seem to overcome the RGMa inhibition. Quantification indicates HFE2 significantly reduces the RGMa induced inhibition of growing axons. (*P<0.001).

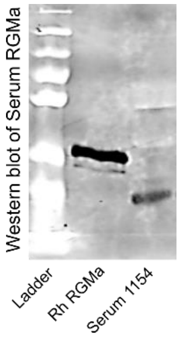

Supplementary Fig. 8 | RGMa Western Blot in human serum. Rhesus Monkey (Rh) RGMa was cloned with a myc-His Tag on its C-terminal part, expressed in HEK-293 cells, purified on Ni-agarose, and used as a positive control. RhRGMa and human serum (serum 1154) were loaded and blotted with an anti-RGMa antibody. This shows the presence of RGMa at ~30 kDa in human serum.

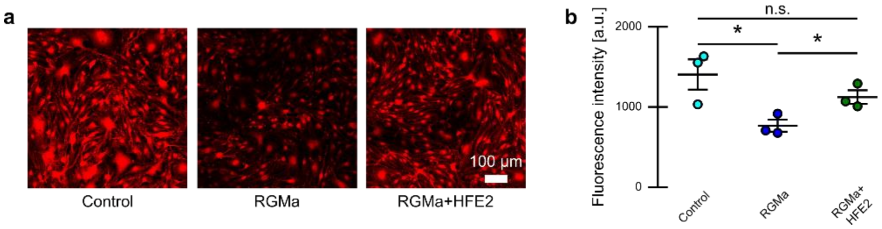

Supplementary Fig. 9 | PDGF-B ICC levels. a, Confocal imaging of PDGF-B in cultured endothelial cells in the presence of vehicle, RGMa, and RGMa+HFE2. **b**, Quantification of fluorescent intensity between control, RGMa, and RGMa+HFE2 (mean ± s.e.m.; unpaired two-tail t-test; replicates n=3). *$P<0.05$. Source data are provided as a Source Data file (includes exact p-values).

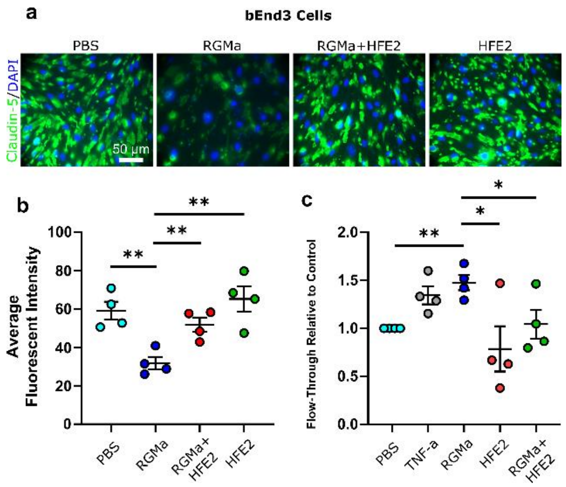

Supplementary Fig.10 HFE2 treatment reverts the RGMa effect on Claudin-5 expression and monolayer flow through. a, Immunocytochemistry of Claudin-5 in bEnd3 cell monolayer after PBS, HFE2, RGMa, RGMa+HFE2, and TNFα treatments. (Scale Bar, 50 µm). **b,** Quantification of claudin-5 immunocytochemistry (mean ± s.e.m.; unpaired two-tail t-test; replicates n=4). Scale bar, 100 µm. **c,** Transwell permeability leakage assay performed on a monolayer of bEnd3 cells using 70kDa FITC-dextran (mean ± s.e.m.; paired two-tail t-test; replicates n=4). *P<0.05, **P<0.01. Source data are provided as a Source Data file (includes exact p-values).

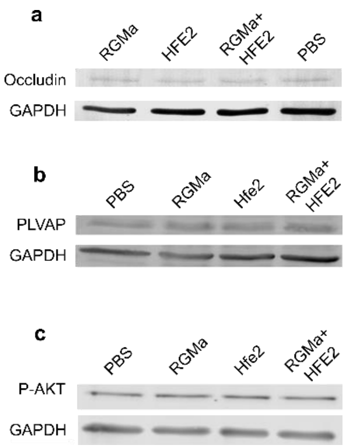

Supplementary Figure 11 | Effect of RGMa and HFE2 treatment on Occludin, PLVAP, and P-AKT expression in brain endothelial cells. Western blotting of bEnd3 cell lysates: **a,** Occuldin (~65kDa), **b,** PLVAP (~55kDa), and **c,** P-AKT (~60kDa) after indicated treatment. Source data are provided at the end of Supplementary Information.

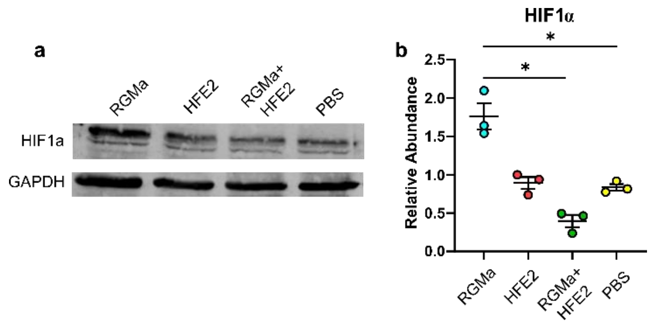

Supplementary Figure 12 | Effect of RGMa and HFE2 treatment on HIF1α expression in brain endothelial cells. a, Western blotting of bEnd3 cell lysates stained with HIF1α (~93kDa) primary antibody after indicated treatment. **b,** Quantification of HIF1α expression relative to control. All conditions normalized to GAPDH (mean ± s.e.m.; paired two-tail t-test, replicates n= 3). *P<0.05. Source data are provided as a Source Data file (includes exact p-values) and at end of Supplementary Information.

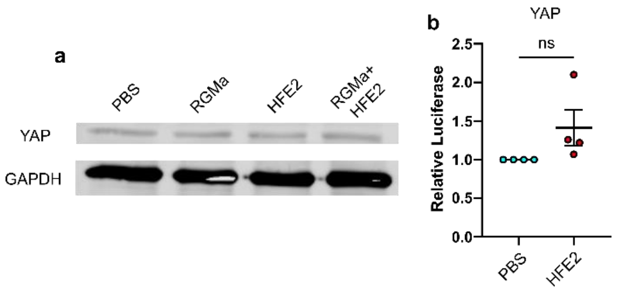

Supplementary Figure 13| HFE2 and RGMa do not affect YAP activity in brain endothelial cells. a, Western blotting of YAP expression (~70kDa) in bEnd3 cell lysates after indicated treatment (mean ± s.e.m.; paired two-tail t-test, replicates n=3) normalized to GAPDH. **b,** Evaluation of Notch luciferase activity in bEnd3 cell lysates after treatment with HFE2 protein for 18 hours. All Data are normalized to control. Source data are provided as a Source Data file (includes exact p-values) and at end of Supplementary Information.

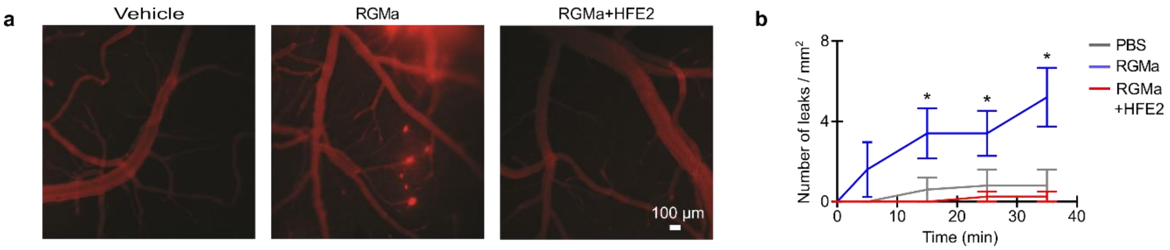

Supplementary Fig. 14 | Protection from RGMa induced BBB disruption by HFE2.
a, Representative widefield BBB leakage patterns of TR-dextran in vehicle, RGMa, and RGMa + HFE2 treated mice. **b**, Quantification of the number of BBB leaks observed in vehicle, RGMa, and RGMa + HFE2 treated mice over time (mean ± s.e.m.; unpaired two-tail t-tests; replicates PBS n=5,

RGMa n=5, and RGMa+HFE2 n=4). *P<0.05. Source data are provided as a Source Data file (includes exact p-values).

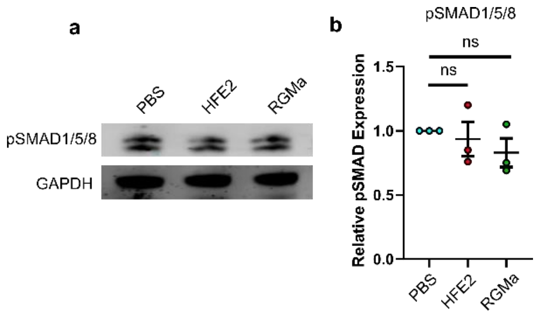

Supplementary Figure 15 | Effect of RGMa and HFE2 treatment on pSMAD1/5/8 expression in brain endothelial cells. a, Western blotting of bEnd3 cell lysates stained with pSMAD1/5/8 (~60kDa) primary antibody after indicated treatment. **b,** Quantification of pSMAD1/5/8 expression relative to control. All conditions normalized to GAPDH (mean ± s.e.m.; paired two-tail t-test, replicates n= 3). Source data are provided as a Source Data file (includes exact p-values) and at end of Supplementary Information.

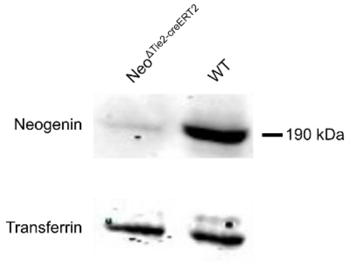

Supplementary Fig. 16 | Inducible knockout of Neogenin expression in endothelial cells. Western blot of Neogenin and Transferrin receptor (reference) in Neo$^{\Delta Tie2\text{-}creERT2}$ mice with (TAM+) and without (TAM-, wide-type) tamoxifen. Source data are provided at end of Supplementary Information.

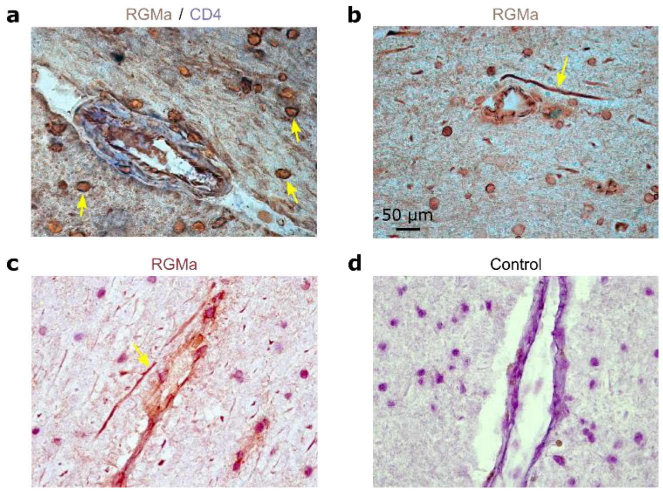

Supplementary Figure 17 | Expression of RGMa in MS lesions. Brain sections from autopsy material of MS patients were stained against RGMa. **a,** RGMa (red deposits) co-localizes with endothelial cells (arrows) with $CD4^{+}$ cells (marker of inflammation, blue deposits, arrowheads) in an MS plaque. RGMa was observed in MS plaques in perivascular and parenchymal areas and in normal appearing white matter. **b,** The blood vessel in the middle shows EC staining and staining of inflammatory cells infiltrating the arterial wall. In addition, RGMa immunostained axons (arrow) were found. **c,** RGMa is found on inflammatory cells infiltrating the wall of the blood vessel seen in normal appearing white matter. In addition, RGMa immunostained axons (arrow) and positive inflammatory cells are seen. **d,** Control of the immunoperoxidase reaction is negative. H&E was used for counterstaining in C and D. H&E is used to stain endothelial cells.

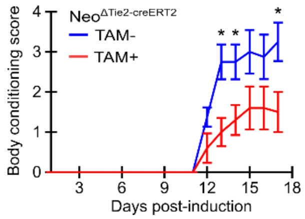

Supplementary Fig. 18 | Effect of endothelial neogenin expression on EAE progression.
Body conditioning scores are shown for days post-EAE induction for $Neo^{\Delta Tie2\text{-}creERT2}$ mice with tamoxifen (TAM+) and without tamoxifen (TAM-) administration. Mice were sacrificed on day 18. Body conditioning assessment (‘0’ no paralysis to ‘4’ forelimb weakness) of EAE-induced mice Tamoxifen treatment a week before EAE induction. (mean ± s.e.m.; unpaired t-tests). *P<0.05. Source data are provided as a Source Data file (includes exact p-values).

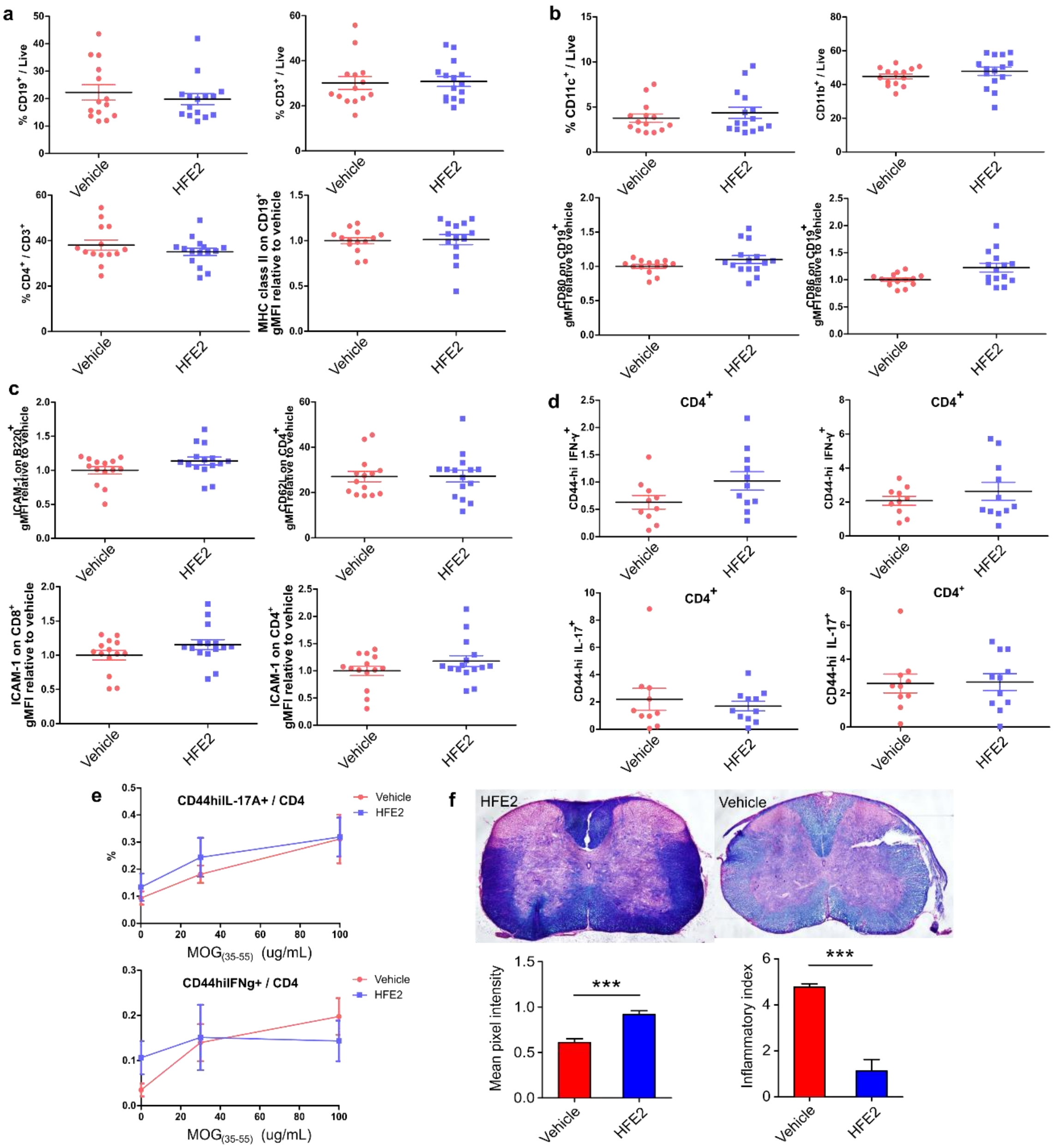

Supplementary Fig. 19 | No immunological effect from protein derived treatments. a-e, Mice were induced with EAE and splenocytes were harvested 10 days post induction (n=15). **a,** Soluble HFE2 has no effect on naïve immune cell populations. Splenocytes were harvested and stained as described. No difference was observed in the percentage of B cells, T cells, or MHC II on B cells in HFE2-treated versus vehicle-treated animals. **b,** Soluble HFE2 treatment has no effect on naïve antigen-presenting cells. Splenocytes were analyzed for the percentage of CD11c (dendritic cell), CD11b (myeloid cell). Soluble HFE2 does not impact the activation of B cells, as seen using co-activation markers CD80 on B cells, CD86 on B cells. **c,** Soluble HFE2 treatment has no effect on the adhesion properties of naïve T and B cells. Splenocytes were analyzed for their levels of ICAM-1 expression on B cell, ICAM-1 expression on CD8+ T cells, ICAM-1 and CD62L expression in CD4+T cells. **d,** Soluble HFE2 has no effect on activated immune cells. Splenocytes were harvested and stimulated using PMA and Ionomycin and analyzed for the expression of activated CD4+T cells secreting IL-17A or IFN-γ. Draining lymph nodes (caudal, sciatic, lumbar) cells were also analyzed as

described. **e,** Soluble HFE2 has no effect on antigen-specific immune cells. CD4+T cell proliferation was measured using CFSE incorportion in splenocytes treated with 0 or 30ug/mL of MOG(35-55), levels of IL17 and IFN-γ in splenocytes pulsed with 0, 30, or 100μg/mL MOG(35-55) were analyzed. **f,** Soluble HFE2 treatment reduces the number of cellular infiltrates and the extent of de-myelination in EAE-induced mice. Mice induced with EAE were trans-cardially perfused 18 days post induction and stained with H&E in combination with Luxol fast blue. Soluble HFE2-treated mice show less de-myelinating foci in cervical spinal cord sections of EAE-induced mice. Soluble HFE2-treated mice possess: a greater mean pixel intensity of Luxol fast blue staining compared to vehicle-treated animals and a decreased inflammatory index (mean ± s.e.m., unpaired two-tail t-test, n=6 per group). ***P<0.001. Source data are provided as a Source Data file (includes exact p-values).

Supplementary Fig. 19 gating strategy:

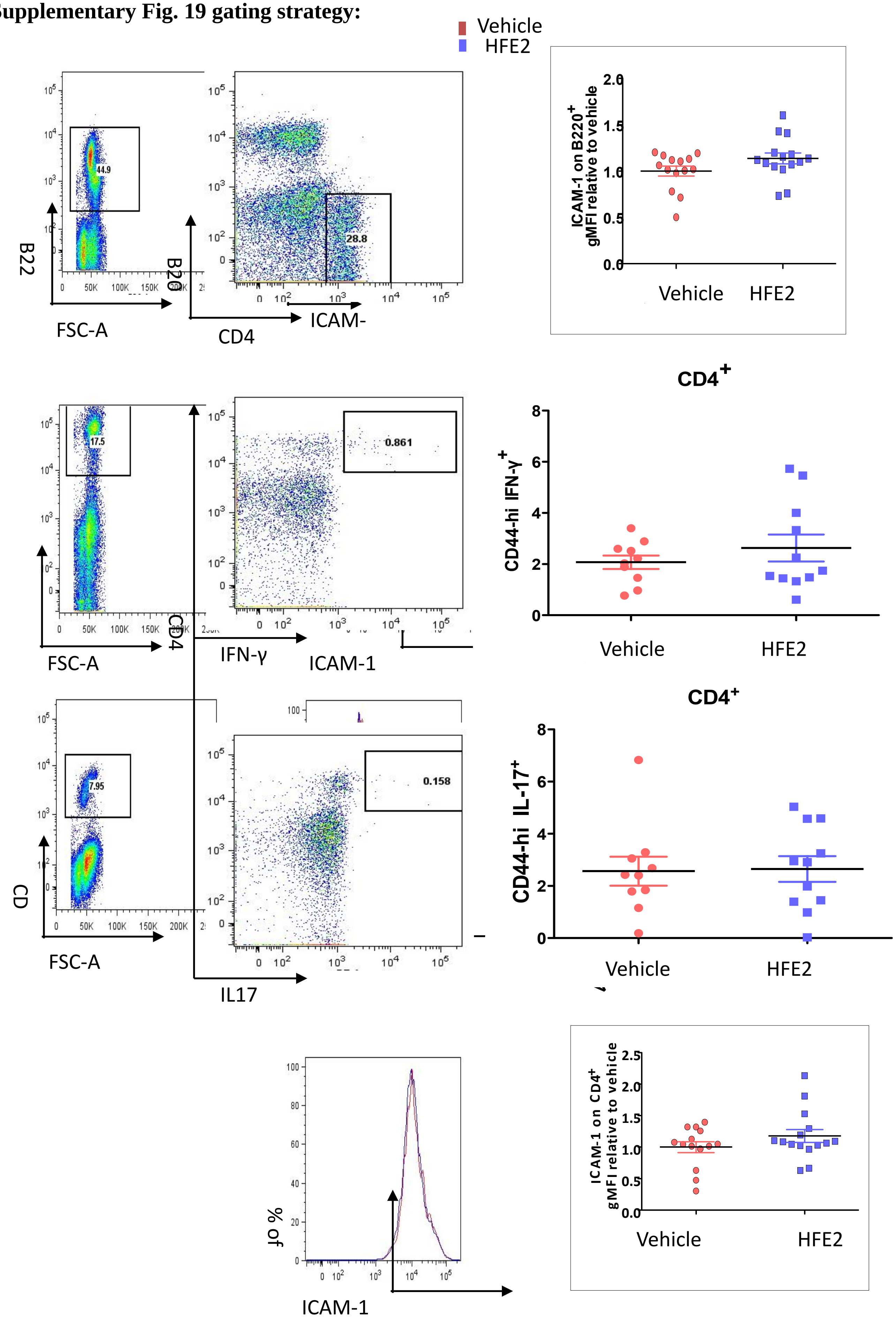

Supplementary Fig. 19 gating strategy:

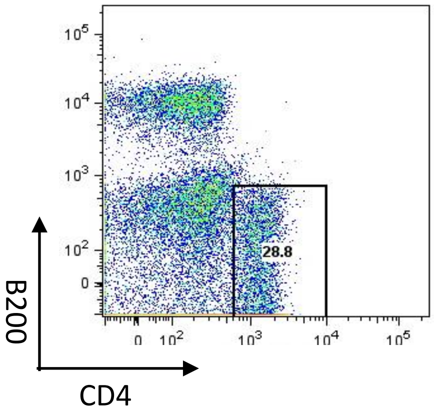

B

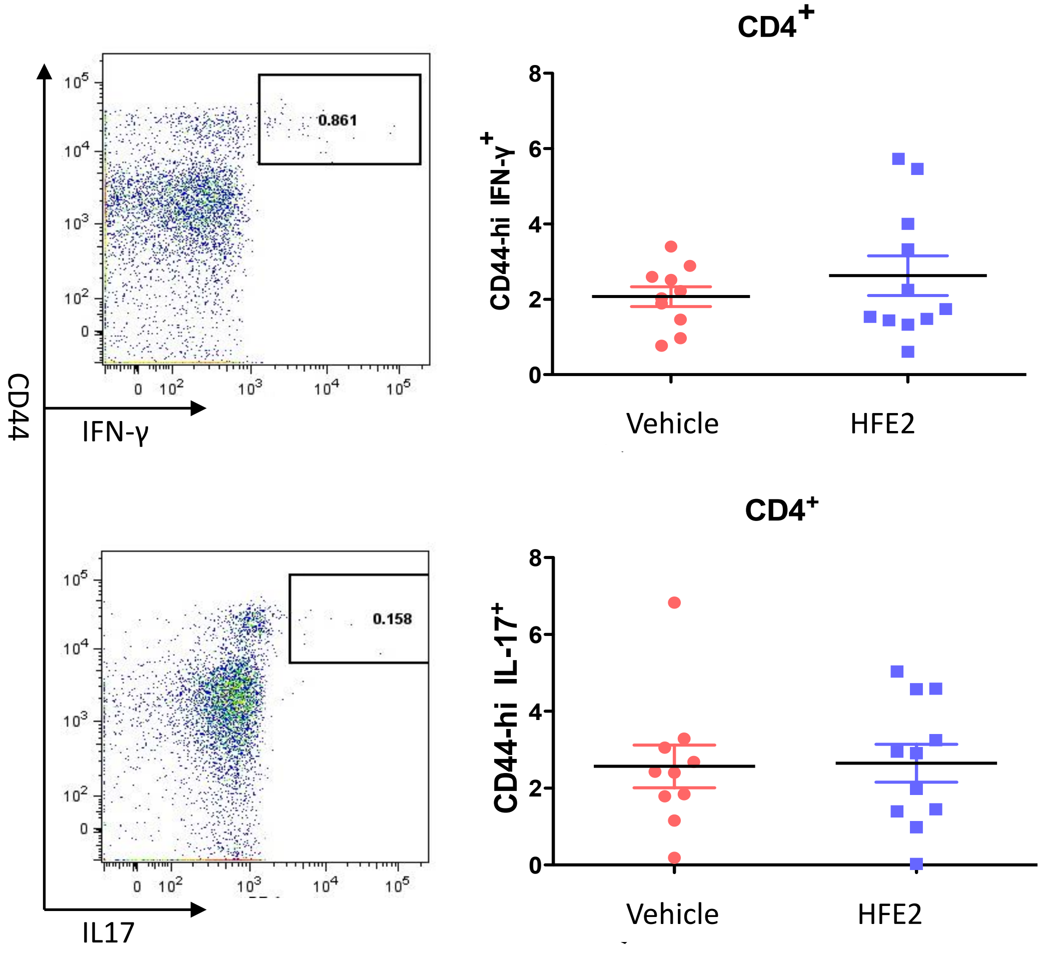

Supplementary Fig. 19 gating strategy:

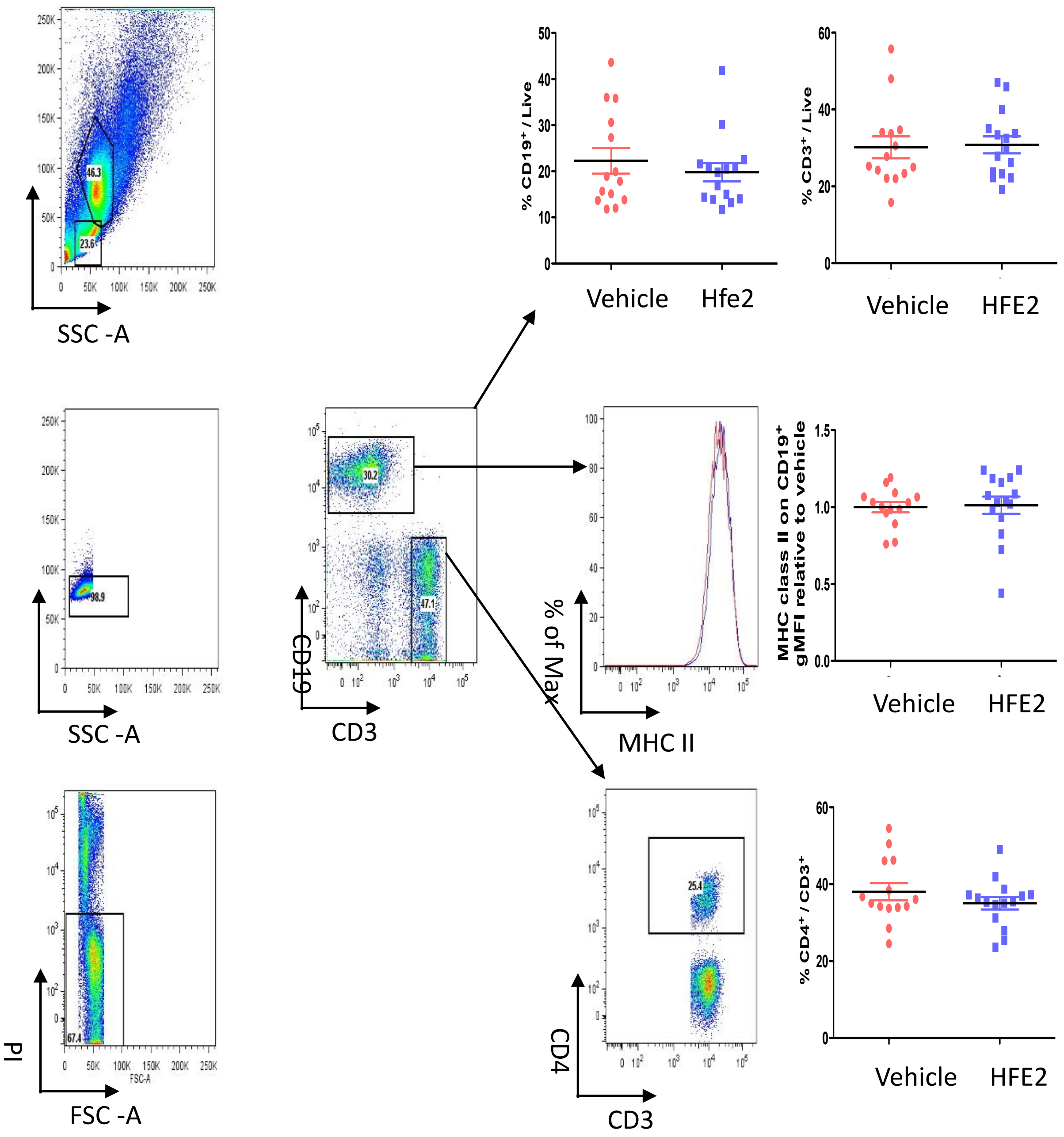

Supplement: Supplementary file 1 — Supplementary Information [file 41467_2024_45303_MOESM1_ESM.pdf]
