# Supplementary material for: The liver and muscle secreted HFE2-protein maintains central nervous system blood vessel integrity
Source: Nat Commun. 2024 Feb 3;15:1037. doi: 10.1038/s41467-024-45303-1 (PMC10838306; doi:10.1038/s41467-024-45303-1)
Supplement: Supplementary file 3 — Description of Additional Supplementary Files [file 41467_2024_45303_MOESM3_ESM.pdf]

Description of Additional Supplementary Files

Supplementary Movie Legends:

Supplementary Movie 1. Liver KO of Hfe2 in mice displays BCB disruption. Representative 3D light sheet imaging of TR-dextran leakage in Hfe2ΔAlb-Cre mice. Corresponds to Extended Fig. 2c. Red channel indicates fluorescent signal from TR-dextran.

Supplementary Movie 2. Light Sheet Imaging of Hfe2fl/fl control shows no BCB disruption. Representative 3D light sheet imaging of TR-dextran leakage in Hfe2fl/fl mice. Corresponds to Extended Fig. 2b. Red channel indicates fluorescent signal from TR-dextran.

Supplementary Movie 3. Multi-photon imaging of brain blood vessels and meninges. Blood vessel in a wild-type mouse are labelled with 70kDa dextran conjugated Texas red. Collagen fibers tracts within the dura matter can be seen using second-harmonic generation (SHG) imaging. Scale bar is 50 µm.

Supplementary Movie 4. Hfe2-deficient mice displays severe BBB disruption. Representative in-vivo multiphoton imaging of TR-dextran leakage over 40 min in Hfe2fl/fl , Hfe2Δalb-cre, AAV8-GFP, and AAV8-Alb-cre mice. Corresponds to Fig. 1c. Red channel indicates regions excluded due to blood vessel labeling, green channel indicates fluorescent signal from TR-dextran, and circular blue region indicates region used for background compensation.

Supplementary Movie 5. Muscle KO of Hfe2 in mice displays BCB disruption. Representative 3D light sheet imaging of TR-dextran leakage in Hfe2ΔActa-Cre mice. Corresponds to Extended Fig. 2d. Red channel indicates fluorescent signal from TR-dextran.

Supplementary Movie 6. Hfe2 and RGMa have opposite effects on BBB integrity. Representative in-vivo multiphoton imaging of TR-dextran leakage over 40 min in RGMa and RGMa+Hfe2 treated brains. Corresponds to Fig. 4b. Same color correspondence as Supplementary Video 2.

Supplementary Movie 7. RGMa disrupts BBB through Neogenin receptor. Representative in-vivo multiphoton imaging of TR-dextran leakage over 40 min in NeoΔTie2-creERT2 mice with (TAM+) and without (TAM-, wide-type) tamoxifen administration. Corresponds to Fig. 5d. Same color correspondence as Supplementary Video 2.
