# Supplementary material for: The liver and muscle secreted HFE2-protein maintains central nervous system blood vessel integrity
Source: Nat Commun. 2024 Feb 3;15:1037. doi: 10.1038/s41467-024-45303-1 (PMC10838306; doi:10.1038/s41467-024-45303-1)
Supplement: Supplementary file 11 — Reporting Summary [file 41467_2024_45303_MOESM11_ESM.pdf]

nature portfolio

Corresponding author(s): Philippe P. Monnier

Last updated by author(s): Nov 1, 2023

# Reporting Summary

Nature Portfolio wishes to improve the reproducibility of the work that we publish. This form provides structure for consistency and transparency in reporting. For further information on Nature Portfolio policies, see our Editorial Policies and the Editorial Policy Checklist.

## Statistics

For all statistical analyses, confirm that the following items are present in the figure legend, table legend, main text, or Methods section.

| n/a | Confirmed | |
|---|---|---|
| ☐ | ☒ | The exact sample size ($n$) for each experimental group/condition, given as a discrete number and unit of measurement |
| ☐ | ☒ | A statement on whether measurements were taken from distinct samples or whether the same sample was measured repeatedly |
| ☐ | ☒ | The statistical test(s) used AND whether they are one- or two-sided<br>*Only common tests should be described solely by name; describe more complex techniques in the Methods section.* |
| ☐ | ☒ | A description of all covariates tested |
| ☐ | ☒ | A description of any assumptions or corrections, such as tests of normality and adjustment for multiple comparisons |
| ☐ | ☒ | A full description of the statistical parameters including central tendency (e.g. means) or other basic estimates (e.g. regression coefficient) AND variation (e.g. standard deviation) or associated estimates of uncertainty (e.g. confidence intervals) |
| ☐ | ☒ | For null hypothesis testing, the test statistic (e.g. $F$, $t$, $r$) with confidence intervals, effect sizes, degrees of freedom and $P$ value noted<br>*Give P values as exact values whenever suitable.* |
| ☒ | ☐ | For Bayesian analysis, information on the choice of priors and Markov chain Monte Carlo settings |
| ☒ | ☐ | For hierarchical and complex designs, identification of the appropriate level for tests and full reporting of outcomes |
| ☒ | ☐ | Estimates of effect sizes (e.g. Cohen's $d$, Pearson's $r$), indicating how they were calculated |

*Our web collection on statistics for biologists contains articles on many of the points above.*

## Software and code

Policy information about availability of computer code

| | |
|---|---|
| Data collection | LI-COR Odyssey, LaVision BioTec ImSpector Pro 5.1, Zen Microscopy 3.3, Micromanager 1.4, Amonlite 1.1 |
| Data analysis | Prism GraphPad 9, ImageJ 1.53, Imaris 9.2, Eclipse 4.19 (Java 20), Apache Commons Math 3.6 |

For manuscripts utilizing custom algorithms or software that are central to the research but not yet described in published literature, software must be made available to editors and reviewers. We strongly encourage code deposition in a community repository (e.g. GitHub). See the Nature Portfolio guidelines for submitting code & software for further information.

## Data

Policy information about availability of data

All manuscripts must include a data availability statement. This statement should provide the following information, where applicable:

- Accession codes, unique identifiers, or web links for publicly available datasets
- A description of any restrictions on data availability
- For clinical datasets or third party data, please ensure that the statement adheres to our policy

Data for all figure grafts are stored and provided in an excel file. Multiphoton videos and light sheet volumetric data are available on request. All other imaging data are available upon request.

# Research involving human participants, their data, or biological material

Policy information about studies with human participants or human data. See also policy information about sex, gender (identity/presentation), and sexual orientation and race, ethnicity and racism.

| | |
|---|---|
| Reporting on sex and gender | Frozen brain tissue specimens acquired at autopsy from six patients with a definitive diagnosis of MS were obtained from the Human Brain and Spinal Fluid Resource Center, Veterans Affairs West Los Angeles Health Care Center., Los Angeles, CA, USA. Case 1- 53y old female, Case 2-53 years old female, Case 3 - 68 y old male, Case 4-62 y old female, Casse 5-47 y old female, Case 6 - 31 y old female.<br>Six control normal brains were obtained in form of normal brain tissue microarray from Biomax (Rockville, MD). Ages/sex of the patients (F, 55, F, 60, F 58, M, 42, F68, F, 62). |
| Reporting on race, ethnicity, or other socially relevant groupings | N/A. |
| Population characteristics | Case 1- 53y old female, Case 2-53 years old female, Case 3 - 68 y old male, Case 4-62 y old female, Casse 5-47 y old female, Case 6 - 31 y old female.<br>Control brains Ages/sex of the patients (F, 55, F, 60, F 58, M, 42, F68, F, 62). |
| Recruitment | Brain tissues were autopsy samples from patients that were diagnose for MS. |
| Ethics oversight | These brain tissue specimens were all autopsy samples from a tissue bank and were exempt from the IRB approval process according to the Department of Health and Human Services Office for Protection from Research Risk Code of Federal Regulations 45 CFR 46.101.6 (4), Exemption No. HR-100201 |

Note that full information on the approval of the study protocol must also be provided in the manuscript.

# Field-specific reporting

Please select the one below that is the best fit for your research. If you are not sure, read the appropriate sections before making your selection.

☒ Life sciences ☐ Behavioural & social sciences ☐ Ecological, evolutionary & environmental sciences

For a reference copy of the document with all sections, see nature.com/documents/nr-reporting-summary-flat.pdf

# Life sciences study design

All studies must disclose on these points even when the disclosure is negative.

| | |
|---|---|
| Sample size | Required sample sizes were estimated a priori using experiments believed to have similar estimated effect sizes. Formal a priori power analysis was not conducted. |
| Data exclusions | Data was only excluded when experimental error produced poor quality images which precluded accurate analysis. |
| Replication | Replication beyond the explicitly shown data was not conducted. The N in each figure presents the number of independent replicate. |
| Randomization | Mice were randomly chosen from litter mate pairs where applicable for group assignment. Genotype groups were assigned after experiments based on PCR identification. In AAV experiments, treatments and viral injections were performed by 2 individuals that had no knowledge of what was administrated. Groups were designed before the animals were submitted to injections by a third person. |
| Blinding | Those collecting data were binded to group identity. Data collection and quantification were performed by different individuals. Analysis metadata (typically segmented regions) used for the quantification of acquired data was archived and independently inspected for quality. Genotype testing was done after experiments to ensure experimenter blinding. In AAV experiments expirimentators were blinded to group allocations and group identities. |

# Reporting for specific materials, systems and methods

We require information from authors about some types of materials, experimental systems and methods used in many studies. Here, indicate whether each material, system or method listed is relevant to your study. If you are not sure if a list item applies to your research, read the appropriate section before selecting a response.

## Materials & experimental systems

| n/a | Involved in the study |
| --- | --- |
| ☐ | ☒ Antibodies |
| ☐ | ☒ Eukaryotic cell lines |
| ☒ | ☐ Palaeontology and archaeology |
| ☐ | ☒ Animals and other organisms |
| ☐ | ☒ Clinical data |
| ☒ | ☐ Dual use research of concern |
| ☒ | ☐ Plants |

## Methods

| n/a | Involved in the study |
| --- | --- |
| ☒ | ☐ ChIP-seq |
| ☐ | ☒ Flow cytometry |
| ☒ | ☐ MRI-based neuroimaging |

# Antibodies

Antibodies used

Hfe2, Goat, polyclonal, R&D, AF3720
CD31, Goat, polyclonal, R&D, AF3628
Neogenin, Rabbit, polyclonal, Santa Cruz, H-175
Neogenin, Goat, polyclonal, Santa Cruz, C-20
Isolectin, Griffonia simplicifolia, Invitrogen, I21411
Fibrinogen, Rabbit, polyclonal, Abcam, ab34269
RGMa, Goat, polyclonal, Santa Cruz, Y-13
RGMa, Goat, polyclonal, R&D, AF2459
Transferrin, Mouse, monoclonal, Santa Cruz, F-8
CD3, Hamster, monoclonal, BioLegend, 145-2C11
B220, Rat, monoclonal, BioLegend, RA3-6B2
CD11b, Rat, monoclonal, BioLegend, M1/70
CD4, Rat, monoclonal, BioLegend, GK1.5
Claudin-5, Rabbit, polyclonal, Abcam, ab15106
GAPHD, Mouse, monoclonal, Abcam, ab8245
CD13, Goat, polyclonal , R&D, AF2335
HIF1a, Rabbit, polyclonal, Invitrogen, 16H4L13
Occludin, Rabbit, polyclonal, Invitrogen, 71-1500
PLVAP, Rabbit, polyclonal, Cell signalling, 38238
YAP, Rabbit, polyclonal, Cell signalling, 4912
P-AKT, Rabbit, polyclonal, Invitrogen, 44-625
pSMAD1/5/9, Rabbit, polyclonal, Cell Signaling, 13820
Donkey anti-goat IgG, Alexa Fluor 488, Invitrogen, A11055
Donkey anti-goat IgG, Alexa Fluor 594, Invitrogen, A11058
Donkey anti-goat IgG, Alexa fluor 647 , Invitrogen, A-21447
Donkey anti-rabbit IgG, Alexa Fluor 594, Invitrogen, A21207
Donkey anti-rabbit IgG, Alexa Fluor 488, Invitrogen, A21206
Goat anti-mouse IgG, IRDye 800, LI-COR, 925-32210
Goat anti-rabbit IgG, IRDye 680, LI-COR, 925-68071
Donkey anti-rat IgG, Alexa Fluor 488, Invitrogen, A21208
Donkey anti-rat IgG, Alexa Fluor 594, Invitrogen, A21209
CD5, 53-7.3, BioLegend
CD3, 145-2C11, BioLegend
CD4, GK1.5, BioLegend
CD8, 53-6.7, BioLegend
CD19, 6D5, BioLegend
CD11b, M1/70, BioLegend
CD11c, N418, BioLegend
CD80, 16-10A1, BioLegend
CD86, GL-1, BioLegend
CD44, IM7, BioLegend
MHC II, M5/114.15.12, BioLegend
CD62L, MEL-14, BioLegend
ICAM-1 (CD54), 3E2, BioLegend
IL-17A, TC11-18H10.41, BioLegend
IFN-γ, XMG1.2, BioLegend

Validation

Western blots were used to validate antibodies against their intended target proteins. Patterns obtained with the antibodies were compared to pattern provided by the manufacturer. Independent validation of primary antibodies was conducted for RGMa and HFE2 which were probed against purified RGMa and HFE2. Neogenin specificity was confirmed in Western Blotting analyses performed on Neo-KO endothelial cells.
Antibodies used in cytometry experiments were all validated by the Wither lab.

## Eukaryotic cell lines

Policy information about cell lines and Sex and Gender in Research

| | |
|---|---|
| Cell line source(s) | HEK-293 and CRL-2299 (bEnd3) cells were obtained from ATCC. |
| Authentication | Both cell lines used were ordered directly from a trusted provider. bEnd3 validated i) by resistance in TEER assay and ii) expression of CD31, a marker for endothelial cells. Further authentication was not done independent of the provider. HEK-293 was only used for protein production. |
| Mycoplasma contamination | Yes, tested with PCR. The cell lines tested negative for mycoplasma. |
| Commonly misidentified lines (See ICLAC register) | HEK293 were used only for protein production. Protein concentration and activity were independently determined. No commonly misidentified lines used. |

## Animals and other research organisms

Policy information about studies involving animals; ARRIVE guidelines recommended for reporting animal research, and Sex and Gender in Research

| | |
|---|---|
| Laboratory animals | C57BL6 wild-type mice or transgenic mice on a C57BL6 background were used for all experiments. Leakage studies used only 6-8 week old mice. Acta -cre mice had a C67B1/129S1 mixed background and were crossed with C57BL6 mice and maintained on a C57Bl6 background.<br>All EAE experiments were done with mice of C57BL6 background (8-10 weeks old). |
| Wild animals | N/A. Only laboratory derived animals were used in this study. |
| Reporting on sex | Both male and female were used in all experimental groups with the exception of our experimental autoimmune encephalomyelitis (EAE) mice which were all female. We did not analyze the effect of biological sex as it was not expected to be a significant factor with respect to BBB in our animal models. |
| Field-collected samples | N/A. No samples were collected outside of the laboratory or outside of a pathologists clinic. |
| Ethics oversight | Animal Care Committee (University Health Network) |

Note that full information on the approval of the study protocol must also be provided in the manuscript.

## Clinical data

Policy information about clinical studies

All manuscripts should comply with the ICMJE guidelines for publication of clinical research and a completed CONSORT checklist must be included with all submissions.

| | |
|---|---|
| Clinical trial registration | N/A. This study is not directly affiliated with any clinical trial. |
| Study protocol | N/A. This study is not directly affiliated with any clinical trial. |
| Data collection | Postmortem tissue collected by pathologists or primary human endothelial cells collected from temporal lobectomy. |
| Outcomes | N/A. No outcomes were tracked for the purposes of this study. |

## Plants

| | |
|---|---|
| Seed stocks | N/A. |
| Novel plant genotypes | N/A. |
| Authentication | N/A. |

# Flow Cytometry

## Plots

Confirm that:

- [x] The axis labels state the marker and fluorochrome used (e.g. CD4-FITC).
- [x] The axis scales are clearly visible. Include numbers along axes only for bottom left plot of group (a 'group' is an analysis of identical markers).
- [ ] All plots are contour plots with outliers or pseudocolor plots.
- [ ] A numerical value for number of cells or percentage (with statistics) is provided.

## Methodology

| | |
|---|---|
| Sample preparation | Described in first paragraph of methods section "Flow Cytometry and Intracellular Cytokine Staining". |
| Instrument | BD LSRII or FACS CantoII cytometer (BD Biosciences). |
| Software | Flowjo software (Tree Star Inc.). No custom code was used. |
| Cell population abundance | Analysis of naive cells, 5x10^5 RBC depleted splenocytes and lymph node cells. For each experiment, at least 100,000 live events were acquired and analyzed. |
| Gating strategy | FSC-A/SSC-A : 25-70K/0-45K; SSC-W/SSC-A : 50-90K/10-110K; PI/FSC-A : 0-11K/0-100K |

- [ ] Tick this box to confirm that a figure exemplifying the gating strategy is provided in the Supplementary Information.
